# The gender gap in outpatient care for non-communicable diseases in Mexico between 2006 and 2022

**DOI:** 10.1186/s41256-024-00377-8

**Published:** 2024-09-29

**Authors:** Edson Serván-Mori, Ileana Heredia-Pi, Carlos M. Guerrero-López, Stephen Jan, Laura Downey, Rocío Garcia-Díaz, Gustavo Nigenda, Emanuel Orozco-Núñez, María de la Cruz Muradás-Troitiño, Laura Flamand, Robyn Norton, Rafael Lozano

**Affiliations:** 1 Center for Health Systems Research, The National Institute of Public Health of Mexico, Universidad Av. 655, 62100 Cuernavaca, Morelos Mexico; 2 The George Institute for Global Health, UNSW, Sydney, NSW Australia; 3 The George Institute for Global Health, School of Public Health, Imperial College London, London, UK; 4 Center for Health Economics and Policy Innovation, Business School, Imperial College London, London, UK; 5 Department of Economics, Tecnologico de Monterrey, Monterrey, Nuevo Leon Mexico; 6 Faculty of Nursing and Midwifery, The National Autonomous University of Mexico, Mexico City, Mexico; 7 Coordinator of Sociodemographic and Prospective Studies, The National Population Council of Mexico, Mexico City, Mexico; 8 Center for International Studies, El Colegio de Mexico, Mexico City, Mexico; 9 Institute for Health Metrics and Evaluation, University of Washington, Seattle, USA; 10 School of Medicine, The National Autonomous University of Mexico, Mexico City, Mexico

**Keywords:** Gender gap, Health service utilization, Segmentation, Outpatient health services, Non-communicable diseases, Universal health coverage, Mexico

## Abstract

**Background:**

Equitable health service utilization is key to health systems' optimal performance and universal health coverage. The evidence shows that men and women use health services differently. However, current analyses have failed to explore these differences in depth and investigate how such gender disparities vary by service type. This study examined the gender gap in the use of outpatient health services by Mexican adults with non-communicable diseases (NCDs) from 2006 to 2022.

**Methods:**

A cross-sectional population-based analysis of data drawn from National Health and Nutrition Surveys of 2006, 2011–12, 2020, 2021, and 2022 was performed. Information was gathered from 300,878 Mexican adults aged 20 years and older who either had some form of public health insurance or were uninsured. We assessed the use of outpatient health services provided by qualified personnel for adults who reported having experienced an NCD and seeking outpatient care in the 2 weeks before the survey. Outpatient service utilization was disaggregated into four categories: non-use, use of public health services from providers not corresponding to the user’s health insurance, use of public health services from providers not corresponding to the user’s health insurance, and use of private services. This study reported the mean percentages (with 95% confidence intervals [95% CIs]) for each sociodemographic covariate associated with service utilization, disaggregated by gender. The percentages were reported for each survey year, the entire study period, the types of service use, and the reasons for non-use, according to the type of health problem. The gender gap in health service utilization was calculated using predictive margins by gender, type of disease, and survey year, and adjusted through a multinomial logistic regression model.

**Results:**

Overall, we found that women were less likely to fall within the “non-use” category than men during the entire study period (21.8% vs. 27.8%, *P* < 0.001). However, when taking into account the estimated gender gap measured by incremental probability and comparing health needs caused by NCDs against other conditions, compared with women, men had a 7.4% lower incremental likelihood of falling within the non-use category (*P* < 0.001), were 10.8% more likely to use services from providers corresponding to their health insurance (*P* < 0.001), and showed a 12% lower incremental probability of using private services (*P* < 0.001). Except for the gap in private service utilization, which tended to shrink, the others remained stable throughout the period analyzed.

**Conclusion:**

Over 16 years of outpatient service utilization by Mexican adults requiring care for NCDs has been characterized by the existence of gender inequalities. Women are more likely either not to receive care or resort to using private outpatient services, often resulting in catastrophic out-of-pocket expenses for them and their families. Such inequalities are exacerbated by the segmented structure of the Mexican health system, which provides health insurance conditional on formal employment participation. These findings should be considered as a key factor in reorienting NCD health policies and programs from a gender perspective.

**Supplementary Information:**

The online version contains supplementary material available at 10.1186/s41256-024-00377-8.

## Background

The burden of non-communicable diseases (NCDs) is rapidly increasing in low- and middle-income countries (LMICs) [1–9]. Its impact on the financial sustainability of their health systems [8, 9] and the NCD-associated costs stemming from high levels of social inequality [10] requires that LMICs reorient the actions of their health systems towards effectively combating NCDs [2, 7, 9, 11].

Equal access to effective health services is a pillar of health system performance [12–14] and critical from a human right perspective on health [15–17]. Access is achieved when individuals recognize their health needs and demand care. It also requires that health services meet several fundamental conditions, such as being geographically available, well-organized, affordable, of good quality, and acceptable. People must be able to access healthcare facilities to benefit from the supply of services, regardless of socioeconomic condition, ethnic status, gender, or place of residence [18]. The failure to meet these conditions leads to suboptimal and inequitable service utilization [1], unfavorable health outcomes [19], and loss of social wellbeing [20]. Within this context, utilization is intrinsically linked to effective access [21].

Before LMICs can address the barriers to accessing health services [22], they must fully understand their social and systemic determinants and pathways [23]. Despite its importance as a health determinant [24], studies in these countries have largely ignored gender when analyzing the effective use of health services for the care of people with NCDs [23, 25]. According to The Canadian Institutes of Health Research, unlike biological sex, *gender refers to the socially constructed roles, behaviors, expressions, and identities of girls, women, boys, men, and gender-diverse people. It influences how people perceive themselves and each other, how they act and interact, and the distribution of power and resources in society* [26]. In highly unequal contexts, such as those prevailing in LMICs, gender is rooted in an environment marked by an asymmetric exercise of power and fundamental rights as well as institutionalized disparities in men’s and women’s access to and use of social services [23, 27].

Gender not only influences how men and women work, perceive diseases, and contract illnesses, but also determines how they seek, access, and use health services, receive care, and are treated by health systems [28]. Despite its relevance, research on health service utilization in LMICs [1, 29–31] has assessed these differences exclusively by sex, overlooking their intersection with multiple social vulnerabilities [32], and thus underestimating the health-related disparities associated with gender [25]. Gender is a critical factor in health status, care access and utilization, and care quality. However, gender comparisons are incomplete without considering the underlying social and economic conditions [33]. Studies rarely explore the interaction of gender with variables unrelated to biological determinants, such as health insurance coverage, income, the capacity to pay for health services, autonomy in decision-making, the burden of family responsibilities, and care models [27]. Additionally, gender asymmetries in health service use are linked to how health care is organized and coordinated [34, 35].

More in-depth analyses of health system segmentation (“*health coverage of the population by position in the labor market*”) [36] are required to fully understand its impact on meeting health system objectives such as efficiency, effectiveness, equity, and the provision of financial protection to the population [36, 37]. Segmentation denotes an uneven distribution of healthcare benefits among social groups. It occurs where access to essential social services for a substantial proportion of the population is contingent on market forces with the potential to induce institutionalized bias in public systems [37–40]. Constituting an explicit government policy, the health system segmentation simultaneously is a cause and consequence of institutionalized mechanisms of systematic exclusion and structural discrimination. These factors undermine the effective exercise of the right to health, especially for the most vulnerable groups [36–41]. Analyzing segmentation specifically would shed light on health systems' underlying dynamics, including barriers to effectively using their goods and services [42]. Nevertheless, very few empirical studies have addressed health system segmentation in LMICs [43].

Mexico, the 15th largest economy [44] and 10th most populous country in the world (second in Latin America and the Caribbean) [45], is experiencing a large and growing burden of NCDs, with men bearing the brunt, except in the case of cancers, which are more prevalent among women [46, 47]. This is occurring within the context of a society characterized by conservative attitudes regarding gender roles [48] and a segmented health system with a corporatist structure [36, 37]. The latter was reaffirmed in 2020, when the government reformed Article 4 of the Mexican Constitution [37], dividing the population into two groups: individuals linked directly/indirectly to the formal economy, enjoying Social Security coverage (50.2% of its population), and the rest, employed in the informal economy, lacking such coverage from a direct source [49]. To date, studies on the use of NCD-related health services have failed to incorporate in-depth analyses from a gender perspective [29–31, 50, 51] and have overlooked its link with health system segmentation and the role of segmentation in generating and deepening health inequalities and limiting quality of care [36]. Furthermore, while several studies suggest that outpatient care tends to be more discretionary and susceptible to social norms, gender bias, and discrimination, there is limited research on this topic in LMICs [33, 52–54], including Mexico.

Based on these factors, this study examined the evolution of the gender gap in the use of outpatient health services provided by trained personnel (doctors, nurses, and other health professionals) among Mexican adults experiencing NCDs from 2006 to 2022. We hypothesized that segmentation in the Mexican health system exacerbated the gender disparities experienced by women and men in the use of services for NCD care.

## Methods

### Design and study population

We conducted a joint cross-sectional population analysis using data from the 2006, 2011–2012, 2020, 2021, and 2022 waves of the National Health and Nutrition Survey (*ENSANUT* by its initials in Spanish). We excluded data from the National Health Survey (*ENSA* by its initials in Spanish) 2000 and the *ENSANUT* 2018–2019 due to a lack of comparability in measuring our outcome. The methodological characteristics of *ENSANUT* have been published elsewhere [55–59]. The *ENSANUT* protocols (available at https://ensanut.insp.mx/) were approved by the Ethics and Biosafety Committees of the National Institute of Public Health in Mexico.

We analyzed data from 312,509 Mexican adults (20 years and older) who were either covered by some form of public health insurance or lacked health insurance. Information was gathered from the following survey modules: sociodemographic characteristics, self-reported health needs (concerning illnesses or physical injuries from accidents or assaults) in the 2 weeks before the survey, types of health problems, and the search for and use of outpatient health services. After excluding 3.7% of surveyed individuals with incomplete information on the characteristics of interest, we analyzed data from a final sample of 300,878 adults, nationally representing 193.3 and 175.4 million women and men, respectively, during the study period. The sociodemographic and health profiles were similar for the excluded and the analyzed individuals.

### Variables

We assessed the use of outpatient health services provided by qualified personnel (doctors, nurses, and other health professionals) to adults who reported having experienced an NCD (cardiovascular and metabolic diseases and risk factors, neurological disorders, cancer, chronic respiratory problems, chronic renal conditions, mental illnesses, and drug abuse [60]) and seeking outpatient care in the 2 weeks before the survey. This time frame of health service utilization was selected to ensure comparability across the different waves of *ENSANUT* and following the recommendations of the international literature which suggest that although a longer recall period may yield a greater quantity of information, the accuracy of the information decreases as the period increases [61].

We approached health system segmentation in Mexico by disaggregating adults into two user subgroups: (i) those receiving outpatient care from providers under their health insurance schemes, and (ii) those receiving outpatient care from private or public providers not belonging to their health insurance schemes. We instrumentalized this variable into four categories of usage patterns: non-use = 0, use of public health services from providers corresponding to the user’s health insurance = 1, use of public health services from providers not correspond to the user’s health insurance = 2, and use of private health services = 3. We considered gender as a binary variable, since data sources collected gender in this way, without distinguishing non-binary genders.

We also analyzed a relevant set of covariates [30, 50, 51, 62–70]: (i) *at the individual level:* age (20–39, 40–59 and ≥ 60 years), marital status (married/free union, divorced/separated/widowed, and single), employed in the last week (yes = 1, no = 0), head of household (yes = 1, no = 0), schooling (in years), health insurance coverage (none, Social Security or *Seguro Popular de Salud*), reasons for non-use pertaining to supply-side problems (related to opening hours, distance to the health facility, insufficient supplies or personnel, delay in the provision of care, and mistreatment by service providers), and reasons for non-use pertaining to the demand-side factors (personal decision, financial problems, or feeling that care was unnecessary); (ii) *at the household level:* indigenous status (yes = 1, no = 0), identified according to the official guidelines, where households are considered indigenous if the head of household, a spouse and/or an older relative such as a grandmother speaks one of Mexico’s indigenous languages [71, 72], and a standardized index (in tertiles) comprised of factorial assets and housing materials as a measure of socioeconomic status [73], where higher values indicated a larger number of assets and better housing conditions; and (iii) *area of residence:* rural/urban/metropolitan, covered by a social program and ranked according to a factorial social-deprivation index (in tertiles) including municipal access to basic public services, housing conditions and income, where higher values indicated higher levels of municipal social development [74], and designated by geographic region (Pacific-North, Border, Pacific-Central, Center-North, Center, Mexico City and State of Mexico, Pacific-South and the Yucatan Peninsula).

### Statistical analysis

We initially calculated averages and percentages (with a 95% confidence interval [95% CI]) for the previously described covariates by gender, survey year, and the entire period analyzed, as well as for the categories of use and reasons for non-use concerning NCDs and other health conditions ($\overline{\text{NCD} }$). We then estimated the gender gap in health service utilization for NCD care, based on predictive margins and adjusted predictions (*aPred*) in a multinomial logistic regression model, controlling for all the sociodemographic characteristics mentioned above and for survey-year fixed effects. Predictions were formulated by gender (male or female), type of disease (NCD or $\overline{\text{NCD} }$), and survey year. Gender gaps were estimated using the following formula:

$${Gender \, gap}_{t}=\left\{\left[\frac{\left({aPred}_{NCD, Male,t}/{aPred}_{\overline{NCD },Male,t}\right)-1}{\left({aPred}_{NCD,female,t}/{aPred}_{\overline{NCD },female,t}\right)-1}\right]-1\right\}\times 100$$

In this metric, positive values indicated a greater incremental likelihood (percentage) of occurrence for men than for women regarding health service utilization for NCD care and health needs unrelated to NCDs. These gaps and their 95% CIs were computed using a nonlinear combination based on the delta method in the Stata statistical package *nlcom* command [75, 76]. We performed all analyses considering a complex survey design and sampling weights using the *svy* package module [77].

## Results

The study included a greater percentage of men than women between the ages of 20 and 39 years, contrary to our findings for the age group ≥ 60 years (Table 1). The percentage of divorced, separated, or widowed individuals was greater among women (18.1% vs. 7.7%), while the percentage of individuals married or in a free union was greater among men (66.1% vs. 59.3%). Women's participation in the labor market increased considerably during the period analyzed, rising from 29.9% in 2006 to 47.5% in 2022, but remained stable for men at approximately 78%. Although the percentage of heads of household was greater for men (61.4% vs. 22.4%), it grew by as much as 65% for women over the years. Men reported a greater average level of schooling (9.3 vs. 8.9 years); however, the difference diminished towards the end of the period analyzed. Before 2019, *Seguro Popular* coverage was greater among women (9.8% vs. 8.2% in 2006 and 36.7% vs. 30.2% in 2011–2012); conversely, after 2019, the lack of public health-insurance coverage was 65% greater among women. The characteristics pertaining to household and place of residence were similar for men and women: 8% lived in indigenous homes, 40% lived in homes of low or medium socioeconomic level, and 80% lived in urban/metropolitan localities situated in low-marginalization municipalities, 20% of which enjoyed coverage by social programs. Their geographic distribution was also similar.

**Table 1:** Main characteristics of Mexican adults analyzed according to gender, 2006–2022

	2006–2022		2006		2011–2012		2020		2021		2022	
	Female	Male	Female	Male	Female	Male	Female	Male	Female	Male	Female	Male
Population weighted	193,285,494	175,439,218	31,049,866	26,471,190	34,960,029	32,000,379	41,805,418	38,281,590	42,538,225	39,281,789	42,921,876	39,414,352
%	52.4	47.6	54.0	46.0	52.2	47.8	52.2	47.8	52.0	48.0	52.1	47.9
	*Calculated percentage or average [95% CI]*											
Age (in years), %												
20–39	47.2 [46.7, 47.8]	48.4 [47.9, 48.9]	50.5 [49.7, 51.2]	50.3 [49.7, 51.0]	51.5 [50.8, 52.1]	52.0 [51.4, 52.7]	44.9 [43.7, 46.0]	46.4 [45.2, 47.5]	45.5 [44.3, 46.7]	47.6 [46.4, 48.7]	45.6 [44.1, 47.0]	47.1 [45.6, 48.5]
40–59	34.4 [34.0, 34.8]	33.9 [33.5, 34.4]	32.8 [32.3, 33.3]	33.0 [32.5, 33.6]	33.3 [32.8, 33.8]	33.4 [32.8, 33.9]	35.7 [34.8, 36.6]	34.6 [33.5, 35.6]	35.4 [34.5, 36.3]	33.8 [32.8, 34.7]	34.1 [33.0, 35.2]	34.6 [33.2, 36.0]
≥ 60	18.4 [17.9, 18.8]	17.6 [17.2, 18.1]	16.8 [16.2, 17.4]	16.6 [16.0, 17.3]	15.2 [14.7, 15.8]	14.6 [14.1, 15.1]	19.4 [18.4, 20.4]	19.1 [18.1, 20.1]	19.1 [18.2, 20.1]	18.7 [17.7, 19.7]	20.4 [19.1, 21.6]	18.3 [17.2, 19.5]
Marital status, %												
Married/free union	59.3 [58.7, 59.9]	66.1 [65.5, 66.6]	64.0 [63.2, 64.8]	72.4 [71.6, 73.2]	62.6 [61.9, 63.3]	70.0 [69.3, 70.6]	57.2 [56.1, 58.4]	62.5 [61.3, 63.7]	58.3 [57.1, 59.6]	64.4 [63.2, 65.6]	56.1 [54.4, 57.8]	63.8 [62.3, 65.3]
Divorced/separated/widowed	18.1 [17.8, 18.5]	7.7 [7.4, 7.9]	15.8 [15.4, 16.3]	5.2 [4.9, 5.5]	16.8 [16.4, 17.2]	6.4 [6.1, 6.7]	19.3 [18.5, 20.0]	8.8 [8.2, 9.5]	18.7 [17.9, 19.5]	8.7 [8.1, 9.4]	19.2 [18.2, 20.2]	8.0 [7.4, 8.7]
Single	22.6 [22.1, 23.1]	26.3 [25.8, 26.8]	20.2 [19.6, 20.8]	22.4 [21.7, 23.1]	20.6 [20.0, 21.2]	23.6 [23.1, 24.2]	23.5 [22.5, 24.4]	28.6 [27.5, 29.7]	23.0 [21.9, 24.0]	26.9 [25.7, 28.0]	24.7 [23.2, 26.2]	28.2 [26.8, 29.5]
Employed in the last week, %	39.5 [38.9, 40.1]	77.7 [77.2, 78.1]	29.9 [29.2, 30.6]	76.6 [75.9, 77.3]	35.8 [35.1, 36.6]	78.9 [78.3, 79.6]	40.2 [38.8, 41.5]	74.6 [73.5, 75.7]	40.6 [39.4, 41.9]	76.6 [75.5, 77.6]	47.5 [45.9, 49.2]	81.3 [80.2, 82.5]
Head of household, %	22.4 [22.0, 22.9]	61.4 [60.9, 62.0]	15.9 [15.5, 16.4]	67.4 [66.7, 68.1]	19.1 [18.6, 19.6]	65.4 [64.8, 66.1]	23.3 [22.4, 24.2]	57.6 [56.4, 58.9]	25.3 [24.2, 26.4]	60.6 [59.4, 61.8]	26.2 [25.0, 27.4]	58.7 [57.1, 60.2]
Schooling (in years), avg	8.9 [8.8, 9.0]	9.3 [9.2, 9.4]	7.4 [7.2, 7.5]	8.0 [7.9, 8.2]	8.1 [8.0, 8.2]	8.6 [8.4, 8.7]	9.5 [9.3, 9.7]	9.8 [9.6, 10.0]	9.5 [9.3, 9.7]	9.8 [9.5, 10.0]	9.5 [9.3, 9.8]	9.8 [9.6, 10.1]
Ethnicity: indigenous, %	7.6 [6.9, 8.4]	7.8 [7.1, 8.5]	8.5 [7.4, 9.7]	8.8 [7.6, 10.0]	8.7 [7.7, 9.7]	8.7 [7.7, 9.7]	7.6 [5.6, 9.7]	7.9 [5.8, 10.1]	6.3 [4.7, 7.8]	6.3 [4.7, 8.0]	7.5 [5.4, 9.6]	7.6 [5.6, 9.7]
Health insurance, %												
Nothing	47.3 [46.4, 48.3]	48.6 [47.6, 49.6]	51.1 [50.0, 52.2]	53.0 [51.8, 54.2]	23.4 [22.7, 24.2]	29.9 [29.1, 30.7]	53.0 [50.7, 55.2]	53.2 [51.0, 55.4]	53.1 [50.8, 55.5]	52.7 [50.2, 55.2]	52.8 [50.2, 55.3]	52.3 [49.6, 55.0]
Social security	44.5 [43.6, 45.3]	44.7 [43.7, 45.6]	39.1 [37.9, 40.3]	38.9 [37.6, 40.1]	39.8 [38.6, 41.0]	39.9 [38.8, 41.1]	47.0 [44.8, 49.3]	46.8 [44.6, 49.0]	46.9 [44.5, 49.2]	47.3 [44.8, 49.8]	47.2 [44.7, 49.8]	47.7 [45.0, 50.4]
Seguro popular	8.2 [7.7, 8.7]	6.7 [6.3, 7.2]	9.8 [8.9, 10.7]	8.2 [7.4, 9.0]	36.7 [35.6, 37.8]	30.2 [29.1, 31.2]	–	–	–	–	–	–
Socioeconomic level, %												
Low	11.1 [10.4, 11.7]	11.3 [10.6, 11.9]	17.9 [16.6, 19.3]	18.5 [17.1, 19.9]	10.8 [10.0, 11.6]	11.3 [10.4, 12.1]	9.6 [7.8, 11.5]	10.2 [8.2, 12.2]	9.3 [7.8, 10.9]	9.6 [7.9, 11.2]	9.4 [7.3, 11.5]	9.1 [7.1, 11.2]
Middle	30.6 [29.9, 31.4]	29.9 [29.1, 30.7]	37.4 [36.2, 38.7]	36.9 [35.6, 38.2]	32.7 [31.7, 33.8]	32.2 [31.1, 33.2]	27.1 [25.3, 29.0]	26.6 [24.8, 28.5]	28.6 [26.7, 30.6]	27.9 [26.0, 29.8]	29.2 [27.3, 31.2]	28.5 [26.5, 30.5]
High	58.3 [57.3, 59.3]	58.8 [57.8, 59.9]	44.6 [42.9, 46.4]	44.6 [42.8, 46.3]	56.4 [55.0, 57.9]	56.5 [55.1, 58.0]	63.2 [60.5, 65.9]	63.2 [60.4, 66.0]	62.0 [59.2, 64.8]	62.6 [59.7, 65.4]	61.4 [58.4, 64.4]	62.4 [59.3, 65.4]
Area of residence, %												
Rural	20.4 [19.2, 21.7]	20.9 [19.7, 22.2]	20.8 [18.8, 22.9]	21.2 [19.1, 23.3]	20.7 [19.0, 22.5]	21.6 [19.8, 23.4]	21.1 [17.4, 24.8]	21.0 [17.2, 24.7]	19.7 [16.0, 23.4]	20.4 [16.6, 24.2]	19.9 [15.5, 24.4]	20.8 [16.3, 25.3]
Urban	26.9 [25.2, 28.6]	26.5 [24.9, 28.2]	24.3 [22.8, 25.8]	23.6 [22.1, 25.1]	19.0 [17.2, 20.8]	18.5 [16.7, 20.3]	30.2 [25.7, 34.6]	30.3 [25.8, 34.7]	29.5 [25.2, 33.8]	29.0 [24.7, 33.3]	29.3 [23.9, 34.8]	29.0 [23.7, 34.3]
Metropolitan	52.7 [51.1, 54.3]	52.5 [50.9, 54.1]	54.9 [52.9, 56.8]	55.2 [53.2, 57.2]	60.3 [58.2, 62.3]	59.9 [57.9, 62.0]	48.7 [44.1, 53.4]	48.8 [44.1, 53.4]	50.8 [46.2, 55.3]	50.6 [46.1, 55.2]	50.7 [44.9, 56.5]	50.2 [44.5, 55.9]
Social programs coverage, %	21.6 [21.0, 22.1]	21.4 [20.9, 21.9]	18.0 [16.1, 19.8]	17.5 [15.7, 19.3]	11.1 [10.7, 11.5]	11.0 [10.7, 11.4]	25.7 [24.5, 26.9]	25.4 [24.3, 26.6]	25.0 [23.8, 26.2]	25.0 [23.8, 26.1]	25.2 [23.7, 26.7]	24.9 [23.4, 26.3]
Municipality deprivation level, %												
Low	78.0 [76.6, 79.3]	78.8 [77.5, 80.1]	44.3 [41.8, 46.8]	45.3 [42.9, 47.8]	67.9 [65.8, 70.0]	68.9 [66.8, 70.9]	89.6 [86.6, 92.7]	89.7 [86.6, 92.8]	89.2 [85.9, 92.5]	89.4 [86.2, 92.6]	88.0 [84.2, 91.9]	88.2 [84.3, 92.0]
Middle	16.4 [15.1, 17.6]	15.9 [14.6, 17.2]	37.7 [35.2, 40.2]	37.2 [34.8, 39.7]	23.3 [21.2, 25.3]	22.4 [20.4, 24.4]	8.1 [5.4, 10.9]	8.2 [5.4, 11.0]	9.6 [6.4, 12.8]	9.6 [6.4, 12.7]	10.0 [6.4, 13.6]	10.3 [6.6, 14.0]
High	5.7 [5.0, 6.3]	5.3 [4.7, 5.8]	18.0 [16.2, 19.9]	17.5 [15.6, 19.3]	8.8 [7.5, 10.1]	8.7 [7.4, 10.0]	2.2 [0.7, 3.8]	2.1 [0.6, 3.6]	1.2 [0.0, 2.5]	1.0 [0.0, 2.1]	2.0 [0.3, 3.7]	1.6 [0.3, 2.8]
Geographic region,												
Pacific-North	8.9 [8.3, 9.6]	9.7 [9.0, 10.4]	8.7 [7.9, 9.4]	9.4 [8.5, 10.2]	8.8 [8.1, 9.5]	9.6 [8.9, 10.4]	9.1 [6.6, 11.6]	9.9 [7.2, 12.7]	8.9 [6.7, 11.0]	9.5 [7.2, 11.8]	9.2 [6.7, 11.8]	9.9 [7.2, 12.6]
Border	12.5 [11.7, 13.2]	13.2 [12.4, 13.9]	12.3 [11.3, 13.3]	13.4 [12.2, 14.5]	12.2 [11.4, 13.1]	12.9 [12.0, 13.8]	12.5 [9.1, 15.8]	13.1 [9.6, 16.5]	12.6 [9.5, 15.7]	13.3 [10.0, 16.5]	12.7 [10.1, 15.3]	13.3 [10.6, 16.0]
Pacific-Central	11.0 [9.8, 12.2]	11.0 [9.8, 12.1]	11.1 [9.7, 12.4]	10.5 [9.2, 11.8]	11.0 [9.8, 12.3]	11.0 [9.8, 12.2]	11.0 [8.2, 13.8]	11.2 [8.3, 14.0]	11.0 [7.4, 14.6]	11.1 [7.5, 14.6]	10.9 [5.9, 16.0]	11.0 [6.1, 15.8]
Centre-North	12.5 [11.7, 13.4]	12.4 [11.6, 13.2]	12.1 [11.1, 13.1]	11.5 [10.6, 12.4]	12.5 [11.4, 13.5]	12.2 [11.2, 13.2]	12.9 [9.6, 16.1]	12.8 [9.5, 16.1]	12.7 [10.5, 14.9]	12.7 [10.4, 14.9]	12.5 [10.1, 15.0]	12.6 [10.1, 15.1]
Centre	10.3 [9.2, 11.4]	9.9 [8.9, 11.0]	10.2 [9.0, 11.4]	10.0 [8.7, 11.3]	10.6 [9.2, 12.0]	10.2 [8.9, 11.6]	10.3 [7.6, 13.0]	9.9 [7.3, 12.5]	10.2 [6.8, 13.6]	9.8 [6.5, 13.2]	10.3 [6.2, 14.5]	9.9 [5.9, 13.8]
Mexico City and State of Mexico	22.3 [21.2, 23.4]	21.8 [20.7, 22.9]	24.1 [22.0, 26.2]	23.9 [21.8, 26.0]	22.4 [20.7, 24.2]	22.0 [20.3, 23.7]	21.8 [18.4, 25.2]	21.2 [17.8, 24.6]	22.0 [18.7, 25.3]	21.5 [18.2, 24.7]	21.7 [16.5, 26.9]	21.1 [15.9, 26.2]
South Pacific	12.8 [11.7, 13.9]	12.2 [11.1, 13.3]	12.7 [11.5, 13.9]	12.1 [11.0, 13.3]	13.0 [11.7, 14.2]	12.3 [11.1, 13.5]	12.7 [9.3, 16.2]	12.2 [8.8, 15.5]	12.7 [9.3, 16.0]	12.1 [8.9, 15.3]	12.8 [8.7, 16.9]	12.3 [8.4, 16.1]
Peninsula	9.6 [8.9, 10.4]	9.9 [9.1, 10.6]	8.9 [8.0, 9.8]	9.3 [8.3, 10.2]	9.5 [8.5, 10.4]	9.8 [8.8, 10.8]	9.7 [6.8, 12.6]	9.9 [7.0, 12.7]	10.0 [7.4, 12.5]	10.1 [7.6, 12.7]	9.8 [7.1, 12.5]	10.0 [7.2, 12.8]

^a^After the December 2018 elections, the new federal administration dismantled *Seguro Popular* and in its place established the Health Institute for Welfare (*Instituto de Salud para el Bienestar*, or *INSABI*) [36]. Data were drawn from the 2006, 2011–2012, 2020, 2021 and 2022 waves of the Mexican National Health and Nutrition Survey (*ENSANUT* by its initials in Spanish). Estimates were based on the complex survey design and sampling weights

**Fig. 1:**
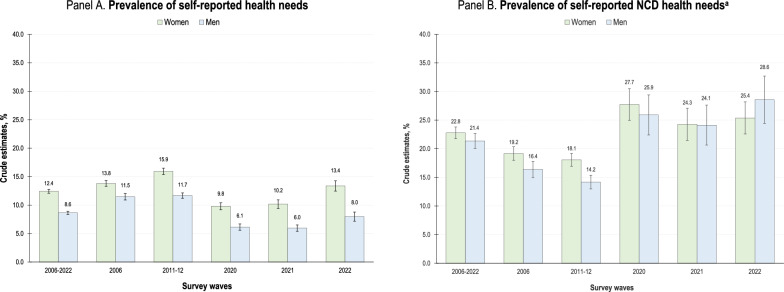
Prevalence of self-reported NCDs and other health needs among Mexican adults according to gender, 2006–2022. ^a^Prevalence estimates for adults who reported a health problem in the 2 weeks prior to the survey. Data drawn from the 2006, 2011–2012, 2020, 2021 and 2022 waves of the Mexican National Health and Nutrition Survey (*ENSANUT* by its initials in Spanish). Estimates based on complex survey design and sampling weights

The percentage of adults reporting health problems in the 2 weeks before the survey was greater among women than among men (12.4% vs. 8.6%, *P* < 0.001) (Fig. 1, Panel A). It peaked in 2011–2012 (15.9% vs. 11.7%, *P* < 0.001) but decreased in 2022 (13.4% vs. 8.0%, *P* < 0.001) (Fig. 1, Panel A). Among adults who reported health needs, the percentage of those presenting at least one NCD was also slightly greater among women during the period of analysis (22.8% vs. 21.4%, *P* < 0.1) (Fig. 1, Panel B). It decreased from 19.2% (vs. 16.4%, *P* < 0.001) in 2006 to 18.1% (vs. 14.2%, *P* < 0.001) in 2011–2012, but then increased and stabilized at approximately 26%, with no differences observed between men and women (Fig. 1, Panel B). Additional estimates (Annex 1) show that, after controlling for all previously described covariates, the relative likelihood of reporting at least one NCD was 23.1% lower in men than in women (relative probability ratio = 0.769, 95% CI: 0.675, 0.877). It increased with age and among those who were not working or had less education.

**Fig. 2:**
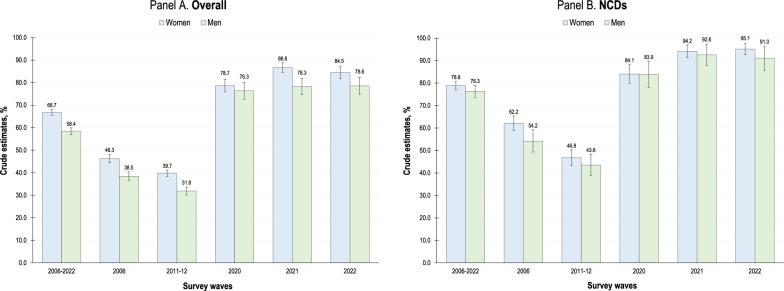
Use of qualified outpatient care by Mexican adults, 2006–2022. Data from the 2006, 2011–2012, 2020, 2021 and 2022 waves of the Mexican National Health and Nutrition Survey (*ENSANUT* by its initials in Spanish). Estimates based on complex survey design and sampling weights

Among those who reported health needs (Fig. 2), the percentage of health service users was greater for women than for men (66.7% vs. 58.4%, *P* < 0.001). This percentage declined from 46.3% (vs. 38.5%) in 2006 to 39.7% (vs. 31.8%, *P* < 0.001) in 2011–12, but then increased significantly to 84.5% (vs. 78.6%, *P* < 0.001) in 2022 (Fig. 2, Panel A). Among those adults who sought NCD care, the percentage of users, which grew towards the end of the period analyzed, was similar for men and women (77%), except in 2006 (62.2% vs. 54.2%, *P* < 0.001). It fell to 45% in 2011–2012 and then increased to approximately 90% in subsequent years (Fig. 2, Panel B).

**Fig. 3:**
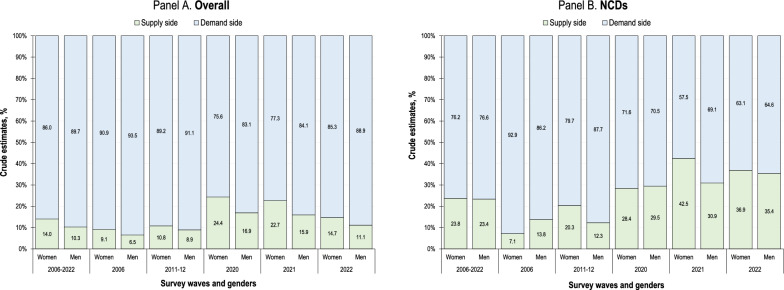
Reasons for non-use of qualified outpatient care by Mexican adults, 2006–2022. Data from the 2006, 2011–2012, 2020, 2021 and 2022 waves of the Mexican National Health and Nutrition Survey (*ENSANUT* by its initials in Spanish). Estimates based on complex survey design and sampling weights

The primary reasons for not using health services were related to the demand-side factors (86.0% in women vs. 89.7% in men) (Fig. 3, Panel A). The percentage was consistently lower for women, with the largest differences observed in 2020 and 2021 (77.3% vs. 84.1% and 85.3% vs. 88.9%, respectively), during which time reasons for non-use referred predominantly to the supply-side issues. Self-reports of these reasons for non-use grew significantly among those who sought NCD care (Fig. 3, Panel B). They increased among women, especially in 2021 when differences were greatest compared to men (42.5% vs. 30.9%).

Estimates from the multinomial logistic regression model showed a growing demand for public health services corresponding to the user’s insurance and an increasing shift of individuals to the private sector (Table 2). Regarding service utilization for NCD care, we found that women were less likely to fall within the non-use category than men (21.8% vs. 27.8%, *P* < 0.001) during the entire study period. Men demonstrated a lower likelihood of using health services from providers corresponding to their insurance than women (47.6% vs. 51.5%), while the use of public health services from providers not corresponding to the user’s insurance and the use of private health services showed minimal differences between women and men (2.6% vs. 2.5% and 24.1% vs. 22.0%, respectively). However, when taking into account the estimated gender gap measured by incremental probability, and comparing health needs related to NCDs against other conditions, men had a 7.4% lower incremental likelihood of falling within the non-use category than women (*P* < 0.001), were 10.8% more likely to use services from providers corresponding to their health insurance (*P* < 0.001), and showed a 12% lower incremental probability of using private services (*P* < 0.001). Conversely, they had an incremental probability 13.5% greater than women of using public services from providers not corresponding to their health insurance (*P* < 0.001). Except for the gap in the use of private services, which tended to decrease, the rest remained stable over the period analyzed (Table 2).

**Table 2:** Gender gap in the use of qualified outpatient services for NCD care, 2006–2022

	2006–2022	2006	2011–2012	2020	2021	2022
	Incremental risk attributable to NCDs, % [95% CI]					
Panel A. Non-use of outpatient health services						
Men						
Non-NCDs (A)	42.8 [40.8, 44.8]	64.8 [62.5, 67.1]	72.9 [71.1, 74.7]	29.8 [26.6, 32.9]	21.3 [18.5, 24.1]	23.3 [20.4, 26.2]
NCDs (B)	27.8 [25.4, 30.2]	47.6 [44.2, 51.0]	58.4 [55.3, 61.6]	18.8 [16.1, 21.6]	12.5 [10.4, 14.6]	13.0 [11.0, 15.0]
Women						
Non-NCDs (C)	35.0 [33.4, 36.6]	57.0 [54.7, 59.3]	66.0 [64.1, 67.8]	23.4 [20.8, 25.9]	16.3 [14.2, 18.4]	18.0 [15.6, 20.3]
NCDs (D)	21.8 [20.0, 23.5]	39.7 [36.7, 42.7]	50.4 [47.5, 53.3]	14.3 [12.3, 16.4]	9.4 [7.9, 10.9]	9.7 [8.3, 11.2]
Gender gap^a^, %	− 7.4 [− 11.2, − 3.6]	− 12.9 [− 17.8, − 8.0]	− 15.9 [− 21.6, − 10.3]	− 4.9 [− 8.5, − 1.2]	− 3.1 [− 6.4, 0.1]	− 3.3 [− 6.1, − 0.5]
Panel B. Non segmented use/use according to public health insurance						
Men						
Non-NCDs (A)	26.6 [25.0, 28.3]	17.6 [16.0, 19.2]	12.2 [11.0, 13.3]	27.7 [24.5, 30.8]	34.0 [30.8, 37.3]	40.5 [37.1, 43.9]
NCDs (B)	47.6 [44.9, 50.3]	35.6 [32.5, 38.7]	26.9 [24.2, 29.6]	48.2 [44.1, 52.4]	55.3 [51.2, 59.3]	62.2 [58.6, 65.8]
Women						
Non-NCDs (C)	30.0 [28.6, 31.5]	21.3 [19.6, 23.1]	15.2 [13.9, 16.4]	30.0 [26.9, 33.1]	35.9 [32.8, 39.1]	43.1 [39.8, 46.4]
NCDs (D)	51.5 [49.0, 53.9]	40.9 [37.9, 44.0]	32.0 [29.4, 34.6]	50.7 [46.7, 54.7]	57.0 [53.1, 61.0]	64.3 [60.9, 67.7]
Gender gap^a^, %	10.8 [4.9, 16.7]	11.8 [6.8, 16.8]	9.4 [5.6, 13.1]	7.5 [1.6, 13.3]	6.2 [− 0.5, 12.9]	8.5 [1.1, 15.9]
Panel C. Segmented use—public/use of another public health provider						
Men						
Non-NCDs (A)	1.6 [1.2, 2.0]	1.8 [1.2, 2.3]	0.7 [0.5, 0.9]	1.8 [1.2, 2.5]	1.9 [1.2, 2.7]	1.7 [1.1, 2.4]
NCDs (B)	2.5 [1.7, 3.4]	3.2 [2.0, 4.3]	1.3 [0.8, 1.8]	2.8 [1.6, 4.0]	2.7 [1.5, 4.0]	2.4 [1.3, 3.5]
Women						
Non-NCDs (C)	1.8 [1.4, 2.1]	2.1 [1.5, 2.7]	0.8 [0.6, 1.1]	1.9 [1.3, 2.6]	1.9 [1.3, 2.6]	1.8 [1.1, 2.4]
NCDs (D)	2.6 [1.9, 3.4]	3.5 [2.4, 4.7]	1.5 [1.0, 2.1]	2.8 [1.7, 4.0]	2.7 [1.6, 3.8]	2.3 [1.3, 3.4]
Gender gap^a^, %	13.5 [2.8, 24.1]	13.9 [5.7, 22.1]	10.7 [5.1, 16.3]	9.4 [0.2, 18.5]	8.2 [− 2.4, 18.8]	11.9 [− 3.3, 27.0]
Panel D. Segmented use—private health provider						
Men						
Non-NCDs (A)	29.0 [27.1, 30.8]	15.9 [14.2, 17.5]	14.2 [12.9, 15.6]	40.7 [37.4, 44.1]	42.8 [39.1, 46.5]	34.5 [30.8, 38.2]
NCDs (B)	22.0 [19.9, 24.1]	13.6 [11.8, 15.5]	13.4 [11.6, 15.1]	30.1 [26.7, 33.6]	29.5 [25.8, 33.2]	22.5 [19.4, 25.5]
Women						
Non-NCDs (C)	33.2 [31.7, 34.7]	19.5 [17.8, 21.3]	18.0 [16.6, 19.5]	44.7 [41.5, 47.9]	45.8 [42.4, 49.3]	37.2 [33.7, 40.7]
NCDs (D)	24.1 [22.0, 26.3]	15.9 [13.9, 17.9]	16.1 [14.2, 18.1]	32.1 [28.6, 35.7]	30.9 [27.1, 34.6]	23.6 [20.5, 26.6]
Gender gap^a^, %	− 12.0 [− 19.4, − 4.6]	− 24.6 [− 41.9, − 7.3]	− 41.4 [− 87.1, 4.4]	− 7.8 [− 14.0, − 1.5]	− 4.8 [− 9.8, 0.3]	− 4.9 [− 9.1, − 0.7]

Regression model adjusted for all characteristics described in Table 1. Estimates based on complex survey design and sampling weights

^a^Calculated according to the following formula: ((((*B*/*A*) − 1)/((*D*/*C*) − 1)) − 1) × 100. Regarding health-service utilization for NCD care, positive values indicate higher probability of occurrence for men than for women. Data from the 2006, 2011–2012, 2020, 2021 and 2022 waves of the Mexican National Health and Nutrition Survey (*ENSANUT* by its initials in Spanish)

## Discussion

The results of this population-based study affirmed the existence of gender inequalities in the use of outpatient care for NCDs by Mexican adults throughout the nearly 2 decades analyzed (2006–2022), structurally determined by the segmentation of the Mexican health system.

During this period, self-reported NCDs in Mexico increased among both genders, consistent with other national, regional, and global estimates [46, 47, 78, 79]. Although previous research has indicated that women utilize health services more frequently than men, it is not consistent across all social groups. Generally, it depends on the type of service required. It is mediated by other factors such as income, age, ethnicity, and place of residence, as well as by variables linked to the type of health financing scheme and the organizational structure of services [27, 80].

The differences in service utilization patterns between men and women reflect three basic trends as follows. First, men and women experience different types of needs, with women having higher rates of morbidity and disability throughout their lives [27, 80]. Furthermore, given their longer life expectancy, women are more likely to experience age-related chronic diseases [27]. Second, how men and women use health services differ, stemming from gender-influenced socialization processes that determine how people recognize symptoms, perceive diseases and seek care [27]. This dynamic is shaped by cultural expectations, including the fact that women more frequently play the role as caregivers. This renders women more adept than men at detecting symptoms, resulting in women being more familiar with formal and informal health-care processes. Differences also exist between men and women regarding their levels of health literacy, defined as “*the ability to obtain, understand, evaluate and use basic health information and services to make well-founded health decisions*” [27, 81]*.* Finally, usage patterns are shaped by structural and institutional factors (less documented) that facilitate or hinder access to health services. These factors are entrenched within existing gender biases in the health-care systems that encourage greater utilization by women through the medicalization of normal biological processes and pregnancy and the availability of financial subsidies for services to be more likely to be used by women. In addition, the structural challenges of health systems include segmentation and fragmentation, which generate inefficiencies in providing care, as well as healthcare financing mechanisms that may favor one gender over the other [27].

In segmented health systems such as the Mexican one, where health insurance coverage is a condition linked to engagement in the labor market (higher labor participation rates result in greater coverage through employment-based schemes) [36], it is important to highlight this trend as a structural enabling factor for the use of health services [24, 82]. To approach the concept of labor market engagement, it is common to use the rate of female participation in the labor market or female economic participation, defined as the total number of employed women aged 15 years or older, as a proportion of the total number of women in that age group, compared with a similar indicator for men [83]. We found that the participation of women in the labor market during the 17 years analyzed increased markedly, rising from 29.9% in 2006 to 47.5% in 2022. This finding aligns with other recent studies in Latin America showing a similar increase in the region over the last 30 years, while statistics for men have remained relatively stable [83]. As a result, the gender gap has narrowed, dropping from almost 36% in 1993 to 23% in 2021, affecting the distribution of household tasks within many families [83]. However, the magnitude of the change varies by country, with Mexico exhibiting one of the lowest female labor participation rates in Latin America (45%), which is below the regional average (53.3%) [83]. Mexico also has one of the lowest rates among countries in the OECD, only surpassing Turkey [35]. Moreover, women experience higher levels of precarious employment and are more likely to work in the informal sector [84]. Despite the positive trend indicating greater female participation in the labor market, recent years have witnessed a slowdown in the rate at which the gender gap is narrowing, a development compounded by the COVID-19 pandemic [83]. The inequality of opportunities between men and women regarding access to the labor market has a profound social impact, imposing high economic costs and consequently preventing the economy from realizing its full growth potential. It is noted that as many as 56% of women in the country work in the informal sector, which further worsens gender inequalities [85]. However, at an individual level, the participation of women in the labor market provides them immediate and long-term benefits, such as financial independence, and the ability to enjoy health insurance coverage and accumulate savings for retirement [83].

Women are more involved than men in household and caregiving duties, such as caring for family members: 40.9% of women versus 14.2% of men provide care for other people [86, 87], with 75.1% of the care being provided by women and 24.9% by men [87]. The amount of time women dedicate to these tasks renders them less available to work in the labor market and participate in other activities. It is one manifestation of gender inequality, which in turn, frequently translates into limited access to healthcare coverage and utilization. In Mexico, it has been documented that, during the COVID-19 pandemic, paid jobs became more precarious. At the same time, the brunt of unpaid work was shouldered mainly by the most vulnerable women (those with low income, living in union and those with children), with regard to both domestic and caregiving work [86]. Previous evidence had shown similar results in Mexico. A detailed analysis of female participation in the Mexican labor market revealed that the traits of discrimination expressed as occupational segregation, wage disparity, benefits and social security, place women in low-skilled and informal activities [88, 89]. For example, in Mexico the probability of women joining the labor market decreases if they are living with a partner, where the man is identified as the head of the household and the provider for the family [88–90].

The previously mentioned gender differences in health service utilization, with women more frequently visiting doctors and making more intense use of hospital services and home health care [27, 80]. However, simple assessments of these differences underestimate gender-associated disparities if they fail to consider underlying health needs [25]. Analyses like ours, which recognize the role of these needs in motivating individuals to seek care, and also take into account the differences in health needs between men and women, show that, among people with similar health profiles, and controlling for observable characteristics, older women are substantially less likely to use outpatient health services than their male counterparts [25]. Additionally, we found that women were 23.1% more likely than men to suffer from at least one chronic condition and this trend intensified with increased age, unemployment, and lower levels of education. This finding is consistent with other studies that have reported that women are more likely to experience multimorbidity (two or more coexisting conditions) [91].

Our study breaks new ground by highlighting how the segmentation of the Mexican health system, which provides differential care for populations depending on whether they are employed in the formal sector of the economy, contributes to accentuating the disadvantages that women experience in the use of outpatient services when faced with health needs related to an NCD. We have documented that men are systematically more likely to receive care in an institution covered by their insurance or in another public institution. In contrast, women are more likely either not to receive care or to use private outpatient services. The segmented Mexican health system conditions the use of health services according to the type of insurance coverage that an individual enjoys, which, in turn, is linked to employment status. The right to health and labor benefits is thus restricted, potentially undermining the effectiveness of the public health policy efforts of the Mexican government, with women suffering the greatest social disadvantages [35].

Although both genders have experienced an increase in the percentage of adults using health services for perceived needs, women continue to be at a disadvantage in receiving care from public institutions. They are more likely to seek care in the private sector, and thus face greater risk of catastrophic out-of-pocket expenditures for them and their families [92, 93]. This suggests that during the process of seeking and using outpatient services, men with NCDs in Mexico enjoy advantages and are more successful in overcoming the barriers imposed by health institutions that provide differential care depending on employment status. Future studies should explore the coping mechanisms that individuals of different genders utilize to eliminate these barriers. Additionally, it is important to consider that our findings indicate that women experience a greater number of NCD-related health needs may reflect the “inverse care law” [94], which states that “*the availability of good health care tends to vary inversely with the need for it in the population served.*” Unfortunately, this law still holds in LMICs [95, 96], where the socially disadvantaged—women in this study—receive less and poorer quality health care despite having a greater number of health needs [95, 96]. This is attributable not only to financial barriers and segmented health systems, but also to social inequalities in seeking care and enjoying financial protection. If we also consider that only 50% of those who seek care for NCDs receive treatment, and of these, only 50% are successfully treated, we can more effectively measure the magnitude of the disadvantages suffered by women in caring for their health related to NCDs [97]. It is imperative to invest in health systems that ensure universal health coverage (UHC) with equity, provided in proportion to need, thus improving the population's health as a whole and reducing health inequalities [95].

These results illustrate the enormous challenges facing LMICs in designing and implementing gender-sensitive health policies, a key mechanism for moving toward UHC, particularly when health insurance highly depends on formal employment participation. The increase in the population aged 60 years or older, especially among women, should inform the design of long-term health policies and programs that take the gender perspective into account. Mexico will have to implement such policies and programs in the context of a health system that has been under tremendous pressure, instituting major reforms over the last 5 years. These developments have deepened the system's segmentation, reversed its decentralization, and generated an operational inability to maintain an adequate supply of medicines. In addition, such pressures have undermined access to health services, adversely affecting the system's financial viability because of reduced public spending on health. This, in turn, has contributed to limiting the supply of public health services and displacing demand toward the private sector [98, 99] among people in all socioeconomic strata.

Our study had several limitations. The first relates to its cross-sectional design, which did not allow for a causal interpretation of the results. Second, the study data were self-reported and may have been subject to recall bias. Nonetheless, previous studies have supported the reliability of self-reports regarding health needs related to chronic diseases [100] and health-care services [101, 102]. In addition, the literature has established that only 50% of those who suffer from these diseases are aware of their diagnosis [97]. Although there is a certain degree of underestimation of health service utilization after long recall periods, there is no evidence that recall biases differ by gender. Hence, our results should probably be considered conservative. Third, we did not evaluate factors related to the characteristics of health service providers, such as professional experience, specialty and available resources, or as geographic and social access [103]. When analyzing the gender gap in health-service utilization, we only partially documented the discrimination that women face in the process of utilizing health care, without considering the degree of evolution or severity of the disease. It has been noted that women suffer sequential and progressive discrimination in seeking care for NCDs in both outpatient and hospital care, among other contexts [25, 104]. Finally, our study did not consider relevant variables concerning attitudes toward specific health problems, which could differ between men and women, and are significantly related to service utilization. These variables include the degree of interest in health, responses to specific symptoms, and family demands [80]. It is clear that women are generally more likely to experience health needs resulting from NCDs than men, in contrast to the differences between men and women regarding the burden of disease due to NCDs [47]. This discrepancy could be explained by differences in the ways in which they conceptualize diseases that are socially constructed in relation to feminine and masculine characteristics and social roles [105].

## Conclusions

We found a notable and persistent gender gap in the use of outpatient services in Mexico. When controlling for health status, women had a greater incremental risk of not using outpatient services and instead using private services for NCD care, indicating a gender-based issue in timely and equitable health-service utilization for women and men. The segmentation of the Mexican health system exacerbates the gender disparities experienced by women in the use of these services and this should be considered in any efforts to reorient health policies and programs towards combating NCDs from a gender perspective.

## Supplementary Information

Additional file 1.

## Data Availability

The analyzed data were obtained from the public repository of the National Health and Nutrition Survey, which is hosted by the National Institute of Public Health (*INSP*) (available at https://ensanut.insp.mx/).

## Abbreviations

NCDs: Non-communicable diseases
UHC: Universal health coverage
LMICs: Low- and middle-income countries
*ENSANUT*: National Health and Nutrition Survey
OCDE: Organization for Economic Cooperation and Development
