# Supplementary material for: The gender gap in outpatient care for non-communicable diseases in Mexico between 2006 and 2022
Source: Glob Health Res Policy. 2024 Sep 29;9:40. doi: 10.1186/s41256-024-00377-8 (PMC11439262; doi:10.1186/s41256-024-00377-8)
Supplement: Supplementary file 1 — Additional file 1. [file 41256_2024_377_MOESM1_ESM.docx]

Supplementary Material 1. **Relative-probability ratios for self-reported health problems among men and women, 2006-2022: multinomial logistic regression model results**

|  | **Overall^a,b^** | |  | **Women^b^** | |  | **Men^b^** | |
| --- | --- | --- | --- | --- | --- | --- | --- | --- |
|  | **Non-NCDs** | **NCDs** |  | **Non-NCDs** | **NCDs** |  | **Non-NCDs** | **NCDs** |
|  | *Relative-probability ratios [SE], base outcome: no health problem in the last two weeks* | | | | | | | |
| Panel A. **Individuals** |  |  |  |  |  |  |  |  |
| Gender (Ref.: Female) |  |  |  |  |  |  |  |  |
| Male | 0.754*** | 0.769*** |  |  |  |  |  |  |
|  | [0.027] | [0.051] |  |  |  |  |  |  |
| Age (Ref.: 20-39 yrs) |  |  |  |  |  |  |  |  |
| 40-59 | 1.049 | 3.169*** |  | 1.034 | 3.207*** |  | 1.056 | 3.014*** |
|  | [0.029] | [0.215] |  | [0.035] | [0.263] |  | [0.045] | [0.357] |
| ≥60 | 1.219*** | 4.808*** |  | 1.185*** | 4.752*** |  | 1.216** | 4.342*** |
|  | [0.046] | [0.374] |  | [0.055] | [0.459] |  | [0.075] | [0.587] |
| Marital status (Ref.: Married/free union) |  |  |  |  |  |  |  |  |
| Divorced/separated/widowed | 0.970 | 1.017 |  | 0.911* | 1.005 |  | 1.106 | 0.960 |
|  | [0.035] | [0.057] |  | [0.040] | [0.071] |  | [0.069] | [0.102] |
| Single | 0.856*** | 0.706*** |  | 0.766*** | 0.701*** |  | 1.024 | 0.623** |
|  | [0.029] | [0.052] |  | [0.034] | [0.062] |  | [0.069] | [0.090] |
| Employed in the last week (Ref.: No) | 0.910** | 0.645*** |  | 1.027 | 0.826** |  | 0.764*** | 0.460*** |
|  | [0.027] | [0.034] |  | [0.036] | [0.055] |  | [0.036] | [0.039] |
| Head of household (Ref.: No) | 1.466*** | 1.350*** |  | 1.478*** | 1.334*** |  | 1.662*** | 1.327* |
|  | [0.039] | [0.066] |  | [0.057] | [0.091] |  | [0.098] | [0.146] |
| Schooling (in yrs), avg | 1.000 | 0.967*** |  | 1.001 | 0.953*** |  | 0.996 | 0.983* |
|  | [0.003] | [0.005] |  | [0.004] | [0.006] |  | [0.004] | [0.008] |
| Panel B. **Household** |  |  |  |  |  |  |  |  |
| Ethnicity: Indigenous (Ref.: No) | 0.899* | 0.818* |  | 0.916 | 0.738** |  | 0.876 | 0.945 |
|  | [0.047] | [0.076] |  | [0.057] | [0.077] |  | [0.067] | [0.128] |
| Health insurance (Ref.: Seguro Popular) |  |  |  |  |  |  |  |  |
| Nothing | 1.062 | 1.135 |  | 1.050 | 1.075 |  | 1.065 | 1.196 |
|  | [0.039] | [0.082] |  | [0.047] | [0.099] |  | [0.052] | [0.128] |
| Social Security | 1.151* | 1.432*** |  | 1.157* | 1.366** |  | 1.140 | 1.533** |
|  | [0.068] | [0.141] |  | [0.080] | [0.149] |  | [0.089] | [0.228] |
| Socioeconomic level (Ref.: Low) |  |  |  |  |  |  |  |  |
| Middle | 1.082 | 1.346*** |  | 1.078 | 1.390** |  | 1.099 | 1.275* |
|  | [0.046] | [0.110] |  | [0.060] | [0.145] |  | [0.067] | [0.145] |
| High | 0.959 | 1.328** |  | 0.960 | 1.358** |  | 0.973 | 1.287* |
|  | [0.047] | [0.122] |  | [0.061] | [0.159] |  | [0.067] | [0.162] |
| Panel C. **Area of residence** |  |  |  |  |  |  |  |  |
| Urban (Ref.: Rural) | 0.899* | 0.923 |  | 0.912 | 0.827* |  | 0.875* | 1.085 |
|  | [0.041] | [0.070] |  | [0.050] | [0.073] |  | [0.051] | [0.116] |
| Metropolitan (Ref.: Rural) | 0.908* | 0.909 |  | 0.928 | 0.855 |  | 0.872* | 0.980 |
|  | [0.041] | [0.071] |  | [0.047] | [0.076] |  | [0.053] | [0.112] |
| Social programs coverage | 0.883 | 0.721** |  | 0.911 | 0.709* |  | 0.853 | 0.751 |
|  | [0.062] | [0.089] |  | [0.075] | [0.098] |  | [0.073] | [0.137] |
| Municipality deprivation level (Ref.: Low) |  |  |  |  |  |  |  |  |
| Middle | 0.939 | 0.827** |  | 0.969 | 0.870 |  | 0.906 | 0.766* |
|  | [0.039] | [0.060] |  | [0.048] | [0.069] |  | [0.051] | [0.083] |
| High | 0.862* | 0.771** |  | 0.899 | 0.814* |  | 0.815* | 0.712* |
|  | [0.053] | [0.075] |  | [0.066] | [0.086] |  | [0.071] | [0.112] |
| Geographic region (Ref.: Pacific-North) |  |  |  |  |  |  |  |  |
| Border | 0.955 | 1.009 |  | 1.058 | 1.010 |  | 0.833* | 1.004 |
|  | [0.054] | [0.102] |  | [0.071] | [0.108] |  | [0.060] | [0.152] |
| Pacific-Central | 1.218** | 1.061 |  | 1.326*** | 1.009 |  | 1.084 | 1.130 |
|  | [0.080] | [0.129] |  | [0.100] | [0.141] |  | [0.093] | [0.172] |
| Centre-North | 1.161** | 1.140 |  | 1.192** | 1.183 |  | 1.130 | 1.051 |
|  | [0.060] | [0.107] |  | [0.072] | [0.120] |  | [0.076] | [0.141] |
| Centre | 1.342*** | 1.377** |  | 1.450*** | 1.416** |  | 1.222* | 1.288 |
|  | [0.089] | [0.167] |  | [0.115] | [0.188] |  | [0.107] | [0.217] |
| Mexico City and State of Mexico | 1.563*** | 1.267* |  | 1.613*** | 1.226* |  | 1.517*** | 1.304* |
|  | [0.085] | [0.122] |  | [0.100] | [0.127] |  | [0.104] | [0.175] |
| South Pacific | 1.382*** | 1.205 |  | 1.384*** | 1.074 |  | 1.401*** | 1.395 |
|  | [0.091] | [0.139] |  | [0.107] | [0.129] |  | [0.133] | [0.268] |
| Peninsula | 1.204** | 1.263* |  | 1.264** | 1.231 |  | 1.149 | 1.308 |
|  | [0.074] | [0.133] |  | [0.092] | [0.139] |  | [0.093] | [0.200] |
| Fixed effects - survey wave (ref.: 2006) |  |  |  |  |  |  |  |  |
| 2011-12 | 1.113* | 1.041 |  | 1.083 | 0.937 |  | 1.004 | 0.963 |
|  | [0.055] | [0.093] |  | [0.060] | [0.100] |  | [0.062] | [0.128] |
| 2020 | 0.579*** | 0.778* |  | 0.586*** | 0.784* |  | 0.456*** | 0.644** |
|  | [0.038] | [0.083] |  | [0.043] | [0.094] |  | [0.039] | [0.106] |
| 2021 | 0.662*** | 0.701** |  | 0.648*** | 0.753* |  | 0.501*** | 0.516*** |
|  | [0.040] | [0.084] |  | [0.043] | [0.097] |  | [0.041] | [0.093] |
| 2022 | 0.864* | 1.052 |  | 0.885 | 1.009 |  | 0.586*** | 1.098 |
|  | [0.061] | [0.119] |  | [0.070] | [0.133] |  | [0.056] | [0.174] |
| Intercept | 0.107*** | 0.013*** |  | 0.098*** | 0.015*** |  | 0.091*** | 0.012*** |
|  | [0.008] | [0.002] |  | [0.009] | [0.003] |  | [0.010] | [0.003] |
| Population weighted | 368,724,712 | 368,724,712 |  | 193,285,494 | 193,285,494 |  | 175,439,218 | 175,439,218 |

*** *p*<0.001, ** *p*<0.01, * *p*<0.05. ^a^ Regression model also adjusted for interactions between gender and survey wave and between health-insurance coverage and survey wave. ^b^ Model also adjusted for interaction between health-insurance coverage and survey wave. Data from the 2006, 2011-12, 2020, 2021 and 2022 waves of the Mexican National Health and Nutrition Survey (*ENSANUT* by its initials in Spanish).
